# Heterologous expression of *Phanerochaete chrysosporium* cellobiose dehydrogenase in *Trichoderma reesei*

**DOI:** 10.1186/s12934-020-01492-0

**Published:** 2021-01-06

**Authors:** Lena Wohlschlager, Florian Csarman, Hucheng Chang, Elisabeth Fitz, Bernhard Seiboth, Roland Ludwig

**Affiliations:** 1 Biocatalysis and Biosensing Laboratory, Department of Food Science and Technology, BOKU–University of Natural Resources and Life Sciences, Muthgasse 18, 1190 Vienna, Austria; 2 Research Division Biochemical Technology, Institute of Chemical, Environmental and Bioscience Engineering, TU Wien, 1060 Vienna, Austria

**Keywords:** Cellobiose dehydrogenase, Catalytic constants, Cofactor occupancy, Glycosylation, Lignocellulose depolymerization, *Phanerochaete chrysosporium*, Redox potential, *Trichoderma reesei*

## Abstract

**Background:**

Cellobiose dehydrogenase from *Phanerochaete chrysosporium* (*Pc*CDH) is a key enzyme in lignocellulose depolymerization, biosensors and biofuel cells. For these applications, it should retain important molecular and catalytic properties when recombinantly expressed. While homologous expression is time-consuming and the prokaryote *Escherichia coli* is not suitable for expression of the two-domain flavocytochrome, the yeast *Pichia pastoris* is hyperglycosylating the enzyme. Fungal expression hosts like *Aspergillus niger* and *Trichoderma reesei* were successfully used to express CDH from the ascomycete *Corynascus thermophilus*. This study describes the expression of basidiomycetes *Pc*CDH in *T. reesei* (*Pc*CDH_*Tr*_) and the detailed comparison of its molecular, catalytic and electrochemical properties in comparison with *Pc*CDH expressed by *P. chrysosporium* and *P. pastoris* (*Pc*CDH_*Pp*_).

**Results:**

*Pc*CDH_*Tr*_ was recombinantly produced with a yield of 600 U L^−1^ after 4 days, which is fast compared to the secretion of the enzyme by *P. chrysosporium*. *Pc*CDH_*Tr*_ and PcCDH were purified to homogeneity by two chromatographic steps. Both enzymes were comparatively characterized in terms of molecular and catalytic properties. The pH optima for electron acceptors are identical for *Pc*CDH_*Tr*_ and *Pc*CDH. The determined FAD cofactor occupancy of 70% for *Pc*CDH_*Tr*_ is higher than for other recombinantly produced CDHs and its catalytic constants are in good accordance with those of *Pc*CDH. Mass spectrometry showed high mannose-type *N*-glycans on *Pc*CDH, but only single *N*-acetyl-d-glucosamine additions at the six potential N-glycosylation sites of *Pc*CDH_*Tr*_, which indicates the presence of an endo-N-acetyl-β-d-glucosaminidase in the supernatant.

**Conclusions:**

Heterologous production of *Pc*CDH_*Tr*_ is faster and the yield higher than secretion by *P. chrysosporium*. It also does not need a cellulose-based medium that impedes efficient production and purification of CDH by binding to the polysaccharide. The obtained high uniformity of *Pc*CDH_*Tr*_ glycoforms will be very useful to investigate electron transfer characteristics in biosensors and biofuel cells, which are depending on the spatial restrictions inflicted by high-mannose N-glycan trees. The determined catalytic and electrochemical properties of *Pc*CDH_*Tr*_ are very similar to those of *Pc*CDH and the FAD cofactor occupancy is good, which advocates *T. reesei* as expression host for engineered *Pc*CDH for biosensors and biofuel cells.

## Background

In the gradual but sustained transition from crude oil towards renewable resources for energy, base- and fine chemicals, and materials, lignocellulosic biomass plays a key role. However, its recalcitrant nature makes the depolymerization and fractionation a challenging process [1, 2]. The application of cellulolytic and ligninolytic fungal enzymes in a concerted manner to achieve controlled lignocellulose depolymerization and separation in e.g. biorefineries, is a promising strategy [3]. The rising of lytic polysaccharide monooxygenase (LPMO) as a major polysaccharide depolymerizing enzyme in fungal secretomes with a high applicability in biomass conversion has also raised the interest into its electron donor cellobiose dehydrogenase (CDH, EC1.1.99.18, CAZy: AA3.1) [4]. The first CDH was identified in the supernatant of white-rot fungus *Phanerochaete chrysosporium* (*Pc*CDH) and was considered to be an oxidase by Westermark and Eriksson in 1974 [5]. Bao et al. later found that this enzyme prefers electron acceptors like cytochrome *c* and DCIP [6]. CDH is a two-domain enzyme consisting of a carbohydrate oxidizing dehydrogenase domain containing a FAD cofactor, which is linked to a heme *b*-containing cytochrome domain serving as an electron shuttle. The enzyme oxidizes cellulose and some hemicellulose degradation products (cellobiose, cello-oligosaccharides, xylo-oligosaccharides, and glucose) to the corresponding lactones thereby taking up electrons [7]. The exact function of CDH was unclear for decades [8, 9] until the discovery of LPMO in 2010 [10]. CDH’s auxiliary function as an electron donor and a possible supplier of hydrogen peroxide to LPMO has been suggested by recent publications [11, 12].

Despite the previous uncertainty on its physiological role, CDH was considered an important enzyme for lactose oxidation, glucose biosensing and biofuel cells [13]. Until 2000, *Pc*CDH was only available by isolating the native enzyme from its fungal producer [14, 15] which does not secrete the enzyme in large quantities (e.g. 66 U L^−1^ [6]). For the production of the enzyme in higher quantities and to be able to perform site-directed mutagenesis for mechanistic studies, a recombinant expression system for CDH is necessary. Li et al. presented the first homologous overexpression of *Pc*CDH in *P. chrysosporium*, reaching 600 U L^−1^ after 9 days in stationary cultures [16]. A three-step purification resulted in a recombinant *Pc*CDH with a RZ-value (A_420_/A_480_) of approximately 0.55 and a specific activity of 8 U mg^−1^, while for isolated native *Pc*CDH a RZ of 0.6 [15] and a higher specific activity of 10.3 U mg^−1^ [6] was reported. Shortly after this work, Yoshida et al. reported the heterologous expression of *Pc*CDH in the yeast *Pichia pastoris* (*Pc*CDH_*Pp*_) in 2001 [17]. After 4 days of cultivation, the expressed enzyme exhibited a volumetric activity of 1800 U L^−1^. *P. pastoris* produces a high cell density in a short time span and carries the eukaryotic machinery for the posttranslational processing of proteins. On the downside, it is known for extensive hyperglycosylation [18], which can alter CDH’s intramolecular electron transfer between the dehydrogenase and cytochrome domain. In contrast, the prokaryotic expression host *Escherichia coli* would not introduce *O-* and *N*-glycans, but successful production of CDH has not been accomplished so far. Only expression of the sole dehydrogenase domain was achieved in *E. coli* [19, 20]. Recombinant production of ascomycetes *Corynascus thermophilus* CDH in *T. reesei* and *Aspergillus niger* [20] indicated that fungal expression hosts could result in a decreased and more uniform glycosylation of recombinant CDH. *Trichoderma reesei* is a well-established host for recombinant protein expression and does not produce a native CDH [21] making it a suitable organism for recombinant CDH production. In 2016, Wang and Lu reported the expression of the *cdh* gene from *P. chrysosporium* in *T. reesei* to study the synergy between CDH and cellulases in vivo, however no yield, purification or characterization of the enzyme was described [22]. In this study, we expressed, purified and characterized *Pc*CDH_*Tr*_ and compared the obtained molecular, kinetic and electrochemical data to native *Pc*CDH and *Pc*CDH_*Pp*_.

## Results and discussion

### Production and purification of *Pc*CDH

For heterologous expression of *Pc*CDH_*Tr*_ in *T. reesei*, the cDNA was cloned into the expression vector pLH_*hph*_nat (Additional file 1: Figure S1). The plasmid contains the strong cDNA1 promoter native to *T. reesei* and the hygromycin B phosphotransferase (*hph*) expression cassette as fungal selection marker. Additionally, it carries the *ori* region and the ampicillin resistance cassette for cloning in *E. coli* [23]. The expression construct was transformed into *T. reesei* strain *Δxyr1*, a derivative from *T. reesei* QM9414 (ATCC 26,921) with deletion of the xylanase regulator 1 (*Δxyr1*). *Xyr1* has been shown to be a major cellulase and xylanase regulator [24] and its removal provides a (hemi)cellulase-reduced background advantageous for protein production [23]. Spore electroporation was chosen as transformation method since this technique has been described as a less time consuming and easier to handle technique than protoplast transformation [25]. Most filamentous fungi like *T. reesei* follow the non-homologous end joining pathway for DNA repair and gene integration over homologous directed recombination [26, 27] resulting in a random integration of the target gene.

We verified the presence of the gene encoding for *Pc*CDH in the genomic DNA by colony PCR for the obtained transformants, which were resistant to the selection marker hygromycin B. The gene was integrated in nine out of nine tested transformants, however, we soon discovered that positive retracing of the gene did not ensure the successful expression of the enzyme. We therefore turned to screening for mitotic stability and protein expression. The first was achieved by growing the transformants on PDA plates without selection pressure for 2 days, followed by transferring a piece of young mycelium back to PDA plates containing hygromycin B. Transformants that maintained their growth were considered to have the plasmid stably integrated into their genome. Screening for protein expression was performed in small-scale shake flasks experiments including one flask with untransformed *T. reesei* strain *Δxyr1* as negative control that exhibited no cytochrome *c* activity in the screening. From two transformants that showed CDH activity with the cytochrome *c* assay after 7 days of cultivation the better one was used for protein production in 1-L Erlenmeyer flasks filled with 200 mL of MA-medium. On day 4, the average cytochrome *c* activity measured in the 8 expression flasks was 700 ± 10 U L^−1^. Since no increase was observed on day 5, the cultures were harvested and pooled for purification. In the pooled, clear supernatant, a volumetric activity of 610 ± 2 U L^−1^ was determined. Li et al. obtained a similar activity (600 U L^−1^) in their homologous overexpression of mutated *Pc*CDH, however only after 9 days [16]. Production of *Pc*CDH_*Pp*_ in *P. pastoris* by Yoshida et al. resulted in a higher volumetric activity of 1800 U L^−1^ after 4 days of cultivation [17].

A two-step purification protocol (Table 1) consisting of hydrophobic interaction chromatography followed by anion exchange chromatography resulted in a homogeneously purified protein as judged by SDS-PAGE. The total amount of pure *Pc*CDH_*Tr*_ obtained from eight shaking flasks was 58 mg (determined via absorbance at 280 nm) with a specific activity of 12.7 U mg^−1^.

**Table 1:** Purification scheme of recombinantly produced *Pc*CDH_*Tr*_

	Volume	Volumetric activity^a^	Protein conc	Total activity^a^	Total protein	Specific activity^a^	RZ	Yield	Purification factor
	[mL]	[U mL^−1^]	[mg mL^−1^]	[U]	[mg]	[U mg^−1^]	[A_420_/A_280_]	[%]	[fold]
Supernatant (filtered)	1250	0.61	0.15^b^	765	186^b^	4.11^b^	0.14	100	1
After HIC (PHE Source)	600	1.12	0.26^b^	670	156^b^	4.29^b^	0.37	88	1.04
After AIEX (Source Q)	24	30.38	4.7^b^	729	113^b^	6.46^b^	0.62	95	1.57
			2.4^c^		58^c^	12.7^c^			

^a^Activity measured with lactose and cytochrome *c* at pH 4.5

^b^Protein concentration measured with Bradford assay

^c^Protein concentration determined via the absorbance at 280 nm and ε_280_ = 156,400 M^−1^ cm^−1^

### Molecular properties and MS analysis

**Fig. 1:**
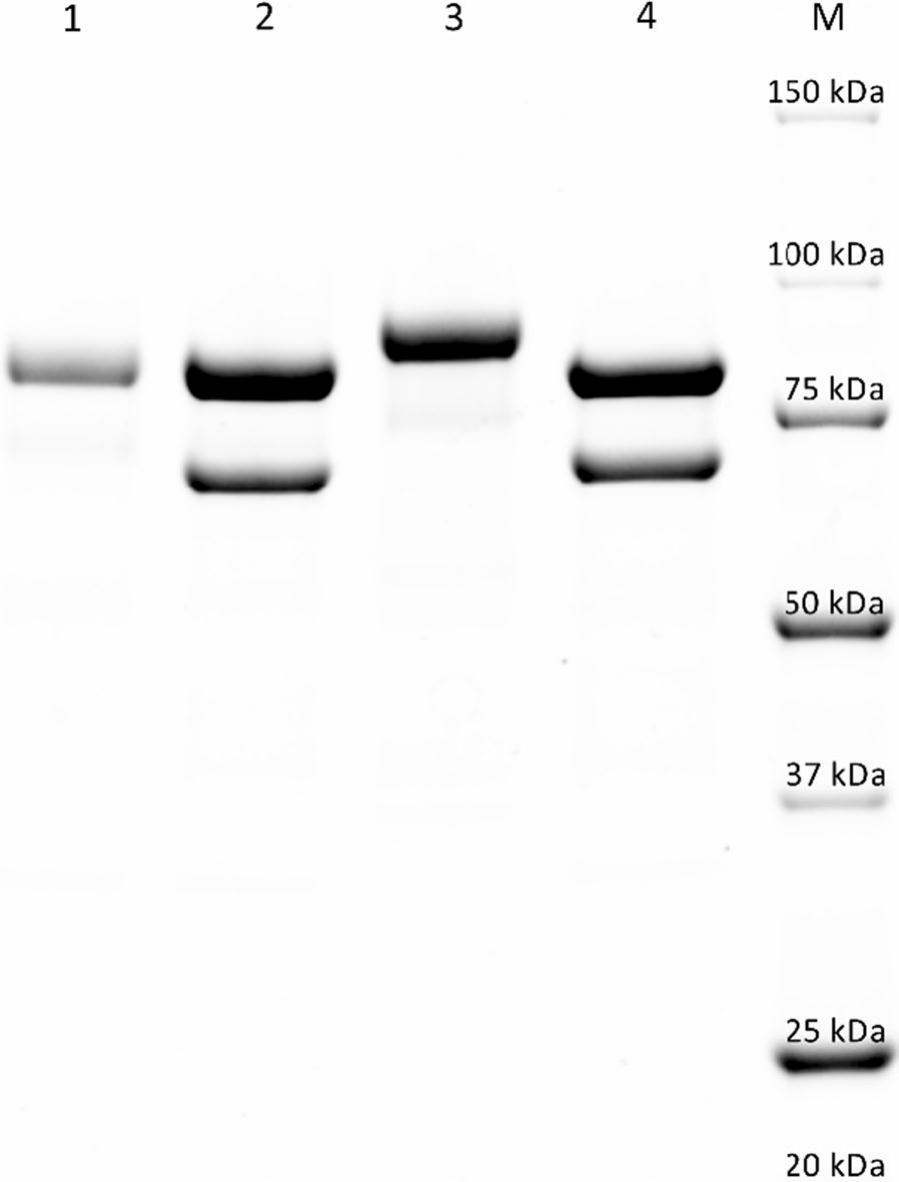
SDS-PAGE of purified and deglycosylated CDHs. *Pc*CDH_*Tr*_ (*Lane 1 Pc*CDH_*Tr*_, *Lane 2* Endo-H_f_ deglycosylated *Pc*CDH_*Tr*_) and native *Pc*CDH (L*ane 3 Pc*CDH isolated from *P. chrysosporium*, *Lane 4* Endo-H_f_ deglycosylated *Pc*CDH). The Precision Plus Protein Dual Color Standard (Bio-Rad) is shown in *Lane M*. Endo-H_f_ has a molar mass of ~ 70,000 g mol^−1^ and appears in Lanes 2 and 4

The high measured RZ-value (A_420_/A_280_) of 0.62 is in accordance with published data of native *Pc*CDH (> 0.6, [15]) as well as recombinant *Pc*CDH_*Pp*_ (0.61, [17]), which verifies the purity of the prepared *Pc*CDH_*Tr*_ and additionally indicates a high occupancy of the heme *b* cofactor in the cytochrome domain. The SDS-PAGE gives a molar mass of 83,000 g mol^−1^ of *Pc*CDH_*Tr*_ (Fig. 1, lane 1). That is around 4000 g mol^−1^ lower than the molar mass of *Pc*CDH with 87,000 g mol^−1^ (Fig. 1, lane 3). The calculated molar mass based on the mature amino acid sequence and the mass of both cofactors is 81550 g mol^−1^. The published molar mass of *Pc*CDH is 90000 g mol^−1^ according to SDS-PAGE analysis [6, 14]. *Pc*CDH_*Pp*_ shows a higher molar mass of 100,000 g mol^−1^, which has been correlated to hyperglycosylation frequently observed for proteins overexpressed in *P. pastoris* [17, 28, 29].

Samples of *Pc*CDH_*Tr*_ and *Pc*CDH were subjected to proteolytic digestion and ESI–MS analysis to determine the *N*-glycosylation on the six NXS/NXT sequons identified as potential glycosylation sites in the mature amino acid sequence of *Pc*CDH (Additional file 1: Figure S2). *Pc*CDH shows high mannose-type *N*-glycans, which was verified for four positions of the isolated enzyme (Additional file 1: Figures S3–S6). Recombinant *Pc*CDH_*Tr*_ only shows the presence of single *N*-acetyl-D-glucosamine (GlcNAc) residues at all six potential glycosylation sites (Additional file 1: Figures S3–S7). These findings coincide with the SDS-PAGE analysis (Fig. 1). The detection of single GlcNAc residues points towards the deglycosylation of the protein-bound high-mannose *N*-glycans. This phenomenon has also been observed for secreted enzymes native to *T. reesei* [30–32]. The three cited articles found that different enzyme production methods (e.g. small-scale shake flasks vs. aerated fermentation) and varying cultivation parameters influence the glycan structures on *T. reesei* cellulases [30]. For example, expression at pH 4 produced Man8 glycans on the cellobiohydrolase I from *T. reesei* while cultivation at pH 5 resulted in cellobioshydrolase I with only GlcNAc residues [31]. These observations were connected to the presence of endo-N-acetyl-β-d-glucosaminidase activity in the supernatant which was later confirmed by Stals et al. who found and characterized the enzyme Endo T [33, 34].

### Spectroscopic analysis (UV/Vis and FAD loading)

**Fig. 2:**
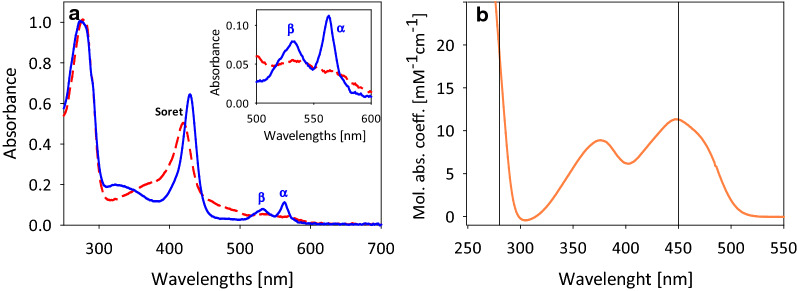
Spectral analysis. **a** UV/Vis spectra of oxidized (red, dashed line) and reduced (blue, solid line) *Pc*CDH_*Tr*_ (70 µM). For obtaining the reduced spectrum, 1 mM cellobiose was added to the purified enzyme solution in 50 mM sodium acetate buffer, pH 4. Inset: Magnification of the spectrum between 500 and 600 nm to show the heme *b* α- and β-peak in more detail. **b** UV/Vis spectrum of the supernatant after trichloroacetic acid precipitation of *Pc*CDH_*Tr*_ for determining the FAD occupancy. The wavelengths 280 and 450 nm are marked with vertical lines and show the contribution of the FAD cofactor to the molar absorption coefficient at 280 nm used for the calculation of the protein concentration

The UV/Vis spectrum obtained for the purified *Pc*CDH_*Tr*_ resembles that of a typical flavocytochrome (Fig. 2a, red dashed line). The dominant Soret-band of the heme *b* cofactor appears at 420 nm. It veils the absorbance of the FAD cofactor, which shows maxima at 375 and 450 nm in its isolated form (Fig. 2b). In the spectrum of the flavocytochrome the presence of the FAD can only be observed in the region between 430–500 nm between the Soret-band and the heme *b* β-peak. Upon reduction, the Soret-band shifts to 429 nm, and the β- and α-peaks of the reduced heme appear at 532 and 563 nm, respectively (Fig. 2a, blue solid line). The recorded spectra of *Pc*CDH_*Tr*_ show all the characteristics typical for published spectra of native *Pc*CDH [6].

For the determination of the FAD loading, a sample of *Pc*CDH_*Tr*_ was subjected to trichloroacetic acid treatment, which resulted in the release of the FAD to the supernatant while precipitating the protein with the heme cofactor. The amount of FAD was calculated from the absorbance at 450 nm in the clarified supernatant, while the protein concentration was determined from the A_280_ value. However, since FAD also absorbs at 280 nm (Fig. 2b) to a non-neglectable extent, its contribution to the A_280_ value was considered. For the initial calculation of protein concentration, 100% FAD loading was assumed and a molar absorption coefficient of ε_280_ = 162.6 mM^−1^ cm^−1^ was used (sum of ε_280_ = 142 mM^–1^ cm^–1^ for *Pc*CDH_*Tr*_ (see Materials and Methods) and ε_280_ = 20.6 mM^–1^ cm^–1^ for free FAD [35]). The resulting FAD occupancy was applied to adapt the percentage of FAD contributing to the overall absorption coefficient at 280 nm. An iterative calculation resulted in a FAD loading of 70% and a molar absorption coefficient for recombinant *Pc*CDH_*Tr*_ of 156.4 mM^–1^ cm^–1^.

This result is similar to the FAD occupancy published for *T. reesei*-produced CDH from *C. thermophilus* (*Ct*CDH) [20]. In contrast, *Ct*CDH expressed in *P. pastoris* contained only 30% FAD in the active site, whereas for native *Ct*CDH 92% occupancy were reported [36]. For native and homologously expressed CDH from *P. chrysosporium* ratios of protein to FAD of 1:1 were reported [6, 16].

### Comparison of specific activities, pH optima and catalytic constants

**Fig. 3:**
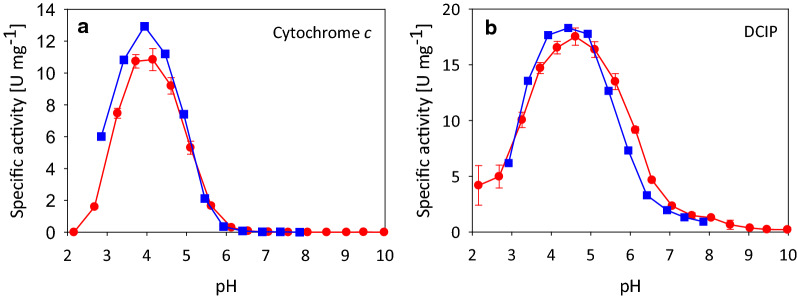
pH Optima. pH Profiles of *Pc*CDH (blue squares) and *Pc*CDH_*Tr*_ (red dots) with the two electron acceptors cytochrome *c* (**a**) and DCIP (**b**). Measurements of recombinant *Pc*CDH_*Tr*_ were done in triplicate. Bars represent standard deviation of the mean. Due to lack of isolated enzyme, measurements of native *Pc*CDH were performed only in duplicate. The deviation of the replicates was in all cases below 5%

*Pc*CDH_*Tr*_ has a pH optimum for the one-electron acceptor cytochrome *c* between 3.5 and 4.0 (Fig. 3a, red dots). For the two-electron acceptor 2,6-dichloroindophenol (DCIP) the pH optimum is found at pH 4.5 (Fig. 3b, red dots). These results are in accordance with the values measured for *Pc*CDH (Fig. 3a, b, blue squares) as well as the published pH optimum for cytochrome *c* (pH 3.5–4.0) determined by Igarashi et al. for *Pc*CDH [14]. The varying pH optima of CDH for different electron acceptors depends on their reduction mechanism. Whereas DCIP is directly reduced at the FADH_2_, the reduction of cytochrome *c* by the heme *b* cofactor depends also on an interdomain electron transfer step between the flavodehydrogenase and the cytochrome domain of CDH, which is also pH dependent. The specific activities of *Pc*CDH_*Tr*_ for cytochrome *c* and DCIP with cellobiose as substrate were 10.8 U mg^–1^ and 17.5 U mg^–1^ at their optimum pH_,_ respectively (Table 2). For the native *Pc*CDH, the specific activities were 12.9 and 18.3 U mg^–1^, respectively, which can be explained by a higher FAD occupancy. The specific activity is also in accordance with the specific activity published for *Pc*CDH by Bao et al. (10.3 U mg^–1^) when considering the different buffer, pH, substrate and electron acceptor concentration [6]. At the respective optimum pH of the electron acceptor, the specific activities were also determined for the electron donor lactose (Table 2).

**Table 2:** Comparison of specific activities of *Pc*CDH_*Tr*_ and *Pc*CDH

	*Pc*CDH_*Tr*_ [U mg^−1^]		*Pc*CDH [U mg^−1^]	
	Cellobiose	Lactose	Cellobiose	Lactose
Cytochrome c	10.8 ± 0.7	15.1 ± 0.3	12.9 ± 0.1	16.0 ± 0.8
DCIP	17.5 ± 0.3	23.4 ± 1.0	18.3 ± 0.6	23.8 ± 1.4

Steady-state kinetic measurements were performed at the pH optimum of the electron acceptor. The determined *K*_M_ and *k*_cat_ values (Table 3) of *Pc*CDH_*Tr*_ for the natural substrate cellobiose are comparable to the catalytic constants published for *Pc*CDH published by Rotsaert et al. [37]. Yoshida et al. also reports similar catalytic constants for *Pc*CDH_*Pp*_ [17]. However, for the recombinant *Pc*CDH dehydrogenase domain produced in *P. pastoris* by Desriani et al. a ten times lower *k*_cat_ (3.6 s^–1^) has been reported [28], which indicates either a low purity of the enzyme preparation of a low cofactor occupancy. The catalytic constants of *Pc*CDH_*Tr*_ for the electron acceptors cytochrome *c* and DCIP are also comparable to the native *Pc*CDH (Table 3).

**Table 3:** Steady-state kinetic constants of *Pc*CDH, *Pc*CDH_*Tr*_ and *Pc*CDH_*Pp*_

Electron donors	This study: *Pc*CDH_*Tr*_^a^			Published: *Pc*CDH				Published: *Pc*CDH_*Pp*_			
	*K*_M_^b^	*k*_cat_^b^	*k*_cat_/*K*_M_^b^	*K*_M_	*k*_cat_	*k*_cat_/*K*_M_	Refs.	*K*_M_	*k*_cat_	*k*_cat_/*K*_M_	Refs.
	[mM]	[s^−1^]	[mM^−1^ s^−1^]	[mM]	[s^−1^]	[mM^−1^ s^−1^]		[mM]	[s^−1^]	[mM^−1^ s^−1^]	
Cellobiose	0.08 ± 0.008	27.8 ± 0.9	340 ± 35	0.04	25.7	643	[32]	0.06^e^	40^e^	689^e^	[16]
				0.11	15.7	143	[14]	0.06	3.6^f^	60.3^f^	[24]
Lactose	1.42 ± 0.08	32.3 ± 1.9	22.7 ± 1.8	1.1	13.4	12	[14]	1.16	4.7^f^	4.1^f^	[24]
Glucose	2109 ± 171	7.8 ± 0.5	0.004 ± 0.0004	1600	2.64	0.0017	[14]				

Electron acceptors	*K*_M_	*k*_cat_	*k*_cat_/*K*_M_	*K*_M_	*k*_cat_	*k*_cat_/*K*_M_	Refs.	*K*_M_	*k*_cat_	*k*_cat_/*K*_M_	Refs.
	[mM]	[s^−1^]	[mM^−1^ s^−1^]	[mM]	[s^−1^]	[mM^−1^ s^−1^]		[mM]	[s^−1^]	[mM^−1^ s^−1^]	
Cytochrome *c*	0.00036 ± 0.00009^c^	13.0 ± 0.2^c^	36,143 ± 8618^c^	0.001	20.5	17,083	[6]	0.001	37.3	25,903	[16]
				0.0007	14.2	20,286	[32]				
DCIP	0.00431 ± 0.00011^d^	24.4 ± 0.2^d^	5655 ± 159^d^	0.004	33	9167	[6]				
				0.006	27	4219	[32]				

^a^Measurements were done in triplicates

^b^Measured with 300 µM DCIP at pH 4.5

^c^Measured with 500 µM cellobiose at pH 4.0

^d^Measured with 500 µM cellobiose at pH 4.5

^e^Ubiquinone used as electron acceptor

^f^Values calculated from V_max_ given in reference [28]

**Fig. 4:**
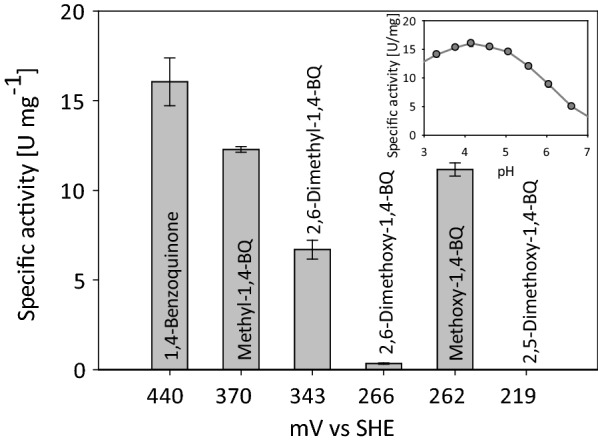
Activity of *Pc*CDH_*Tr*_ with different quinones as electron acceptors. Inset: pH profile of 1,4-benzoquinone (1,4-BQ). Measurements were performed in triplicate. Bars represent the standard deviation of the mean value. Redox potentials were taken from Kracher et al. [33] and recalculated for pH 4.0 using the Nernst equation

Mono- and di-substituted quinones have been shown to be good electron acceptors of CDH and capable redox mediators in the electron transfer system between lytic polysaccharide monooxygenase (LPMO) and glucose dehydrogenase from *Glomerella cingulata*, as CDH a member of the glucose-methanol-choline oxidoreductase family [38, 39]. To study LPMO-related redox mediators as electron acceptors of *Pc*CDH_*Tr*_ the pH optimum pH for unsubstituted 1,4-benzoquinone was determined (Fig. 4, insert). It shows the highest activity with 16.05 U mg^–1^ at pH 4.0, but also exhibits more than 50% activity in a broad pH range between pH 3.0–6.0. The reduction of the substituted quinones under investigation were therefore measured at pH 4.0. The results in Fig. 4 show that with increasing number of electron-donating groups at the quinone and with therefore decreasing redox potential, the reaction rates with *Pc*CDH_*Tr*_ decrease. This indicates that they are less suitable electron acceptors for CDH although they are working well as redox mediators between glucose dehydrogenase and LPMO. When using the midpoint potential of FAD in *Pc*CDH, which was determined by Igarashi et al. to be 106 mV vs. SHE at pH 3.0 and − 132 mV vs. SHE at pH 7.0 [40] and the Nernst equation, the redox potential for pH 4.0 can be calculated (165.5 mV vs. SHE). Comparing this value to the redox potentials of the quinones, it is apparent that all quinones have a higher redox potential than CDH and should be acting as electron acceptors. However, only some of the tested quinones were functional electron acceptors for CDH in this experimental setup. Therefore, we conclude that other factors, such as the steric access of the more bulky quinones to the catalytic site, have a strong rate-limiting effect.

### Redox properties of *Pc*CDH_*Tr*_

**Fig. 5:**
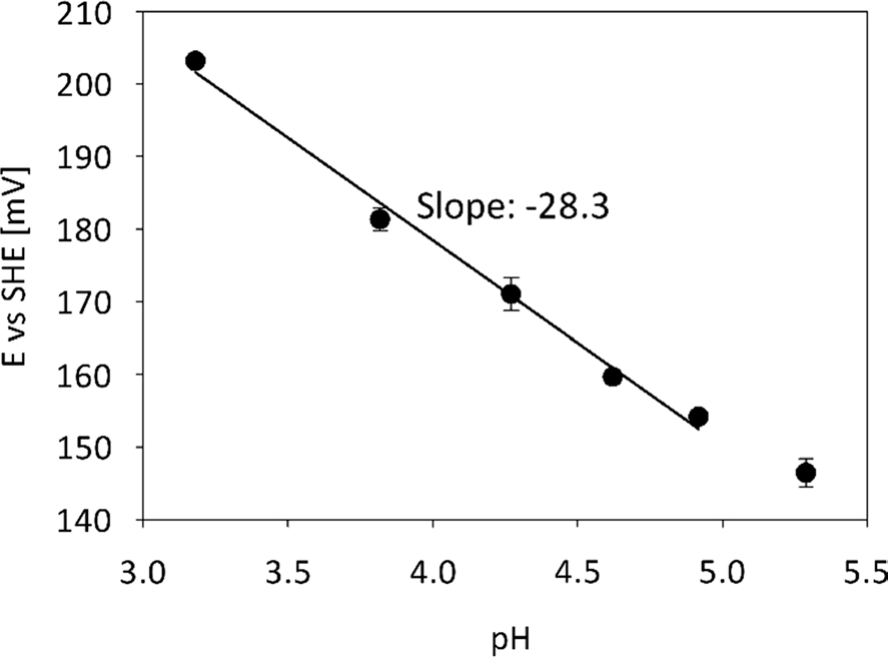
pH-Dependence of the heme-domain redox potential of *Pc*CDH_*Tr*_ obtained by squarewave voltammetry. Midpoint redox potentials were measured using a thioglycerol-modified gold electrode. Ag/AgCl was used as reference electrode, but values were recalculated to SHE. Measurements were done in triplicate

The midpoint potential of the *Pc*CDH_*Tr*_ cytochrome heme-*b* cofactor was determined by square-wave voltammetry. The obtained values range from 203 mV vs. SHE at pH 3.2 to 146 mV vs. SHE at pH 5.3 (Fig. 5). This is in accordance with the redox potential published by Igarashi et al. for *Pc*CDH_*Pp*_ (from 190 mV vs. NHE at pH 3.0 to 160 mV vs. NHE at pH 5.0, which were obtained by cyclic voltammetry [41]). These results demonstrate that *Pc*CDH_*Tr*_ has the same redox properties and can replace *Pc*CDH_*Pp*_ in biosensor and biofuel cell applications.

## Conclusions

The recombinant production, purification and characterization of CDH from *P. chrysosporium* showed that *T. reesei* is a suitable expression host for this flavocytochrome. The heterologous production was efficient by obtaining a high yield in a short cultivation time. The obtained *Pc*CDH_*Tr*_ preparation was pure and had a reasonably high heme *b* and FAD occupancy in the cofactor-binding sites. Especially the lesser degree of glycosylation and the more uniform distribution resulting in less glycoforms make *Pc*CDH_*Tr*_ well suited for applications in which the electron transfer between the domains and further to an electrode surface are required. It will also increase the efficiency and facilitate enzyme immobilization on electrode surfaces. Furthermore, the rediced glycosylation and higher homogeneity of *Pc*CDH_*Tr*_ will be useful for binding and interaction studies on natural substrates of CDH. In terms of catalytic as well as electrochemical properties, *Pc*CDH_*Tr*_ performs in excellent resemblance to native *Pc*CDH. We therefore conclude that *T. reesei* is a suitable expression host for the production of native and engineered basidiomycetous CDHs.

## Materials and methods

### Chemicals, vectors and genes

All chemicals were purchased from Sigma-Aldrich (St. Louis, MO, USA), Carl Roth (Karlsruhe, DE) or VWR (Radnor, PA, USA) in the highest purity available. Reagents for molecular biological methods were obtained from New England Biolabs (NEB, Ipswich, MA, USA). The nucleotide sequence of *Pc*CDH including its native signal peptide was obtained from GenBank (entry U46081.1) that contains the cDNA cloned from *Phanerochaete chrysosporium* strain OGC101 (a derivative of VKM-F-1767) [42]. The coding region of the cDNA flanked by a BclI restriction site on the 5′-end and NotI on the 3′-end for further cloning experiments, was synthesized by BioCat GmbH (Heidelberg, DE) and provided in a pPICZ A plasmid. As expression vector, the plasmid pLH_*hph*_nat (Additional file 1: Figure S1)—a modified version of the plasmid pPcdna1 [23]—was used for transforming and expressing *Pccdh* in *T. reesei*. Purified H_2_O (> 18 MΩ cm) was obtained from a SG system (Barsbüttel, GER) feeded with deionized water.

### Strains and media

For vector construction and amplification, chemically competent *E. coli* strain NEB 5α and methyltransferase-deficient (*dam*^*−*^*/dcm*^*−*^) chemically competent *E. coli *cells were purchased from NEB. *E. coli* cultures were grown at 37 °C in liquid LB (lysogeny broth) medium or on agar-containing LB plates supplemented with suitable selection marker (100 µg mL^–1^ ampicillin, 25 µg mL^–1^ zeocin). Protein expression was carried out in *T. reesei* strain *Δxyr1* [24] that was grown on potato dextrose agar (PDA) plates. Selection and growth of transformants was maintained on PDA plates containing 50 µg mL^–1^ hygromycin B. Expression of *Pc*CDH_*Tr*_ was performed in modified Mandels-Andreotti (MA) medium containing 10 g L^–1^ glucose, 1.4 g L^–1^ (NH_4_)_2_SO_4_, 4.0 g L^–1^ KH_2_PO_4_, 0.3 g L^–1^ urea, 0.3 g L^–1^ MgSO_4_·7H_2_O, 0.4 g L^−1^, CaCl_2_·2H_2_O, 1 g L^−1^ peptone, and 1% (v/v) of trace element solution (0.5 g L^–1^ FeSO_4_·7H_2_O, 0.16 g L^−1^ MnSO_4_·H_2_O, 0.14 g L^−1^ ZnSO_4_·7H_2_O, 0.2 g L^−1^ CoCl_2_·2H_2_O), titrated to pH 6.0 and inoculated with 10^6^ spores mL^−1^. The production of *Pc*CDH by *P. chrysosporium* K3 (a kind gift of the late Prof. Jindřich Volc, Inst. of Microbiology, Czech Academy of Sciences) was done in a medium containing 30 g L^−1^ α-cellulose, 30 g L^−1^ yeast extract and 1 g L^−1^ MgSO_4_·7 H_2_O titrated with H_3_PO_4_ to pH 5.0.

### Construction of *T. reesei* expression vector

The expression vector pLH_*hph*_nat was amplified in *E. coli* strain NEB 5α, purified using the Monarch® Plasmid Miniprep Kit (NEB) and digested with restriction enzymes BglII and NotI from NEB according to their Double Digest Protocol. Plasmid pPICZ A carrying the gene encoding for *Pc*CDH was amplified in methyltransferase-deficient (*dam*^*−*^*/dcm*^*−*^) chemically competent *E. coli *cells because the BclI restriction site on the 5′-terminus is blocked by methylated DNA. Purification of the plasmid was again performed with the Monarch® Plasmid Miniprep Kit and the gene cut from the pPICZ A vector backbone using restriction enzymes BclI and NotI. Since BclI and BglII create the same overhangs, the gene fragment could be ligated into pLH_*hph*_nat using T4 DNA ligase. Successful cloning was verified by agarose gel electrophoreses and DNA sequencing with primers 5pLHseq1 and 3pLHseq1 (Microsynth, Balgach, CH). For transformation, the ligated plasmid was linearized with SbfI.

### Transformation into *T. reesei Δxyr1*

*Trichoderma reesei* transformation was done by spore electroporation according to a modified version of the protocol from [25]. *T. reesei* strain *Δxyr1* was cultivated on PDA plates for 7 days at 30 °C until the plate was fully sporulated. The spores were harvested with sterile, purified H_2_O, filtered through glass-wool to remove mycelium and centrifuged at 3000 × *g* for 3 min at 4 °C. After removing the supernatant, the spores were washed with 1 M 4 °C-cold sorbitol solution twice and resuspended in 100 µL 1 M cold sorbitol solution to obtain a dark-green spore suspension. The linearized plasmid (2.8 µg) was added to 100 µL spore suspension and electroporation carried out in a 0.1-cm electroporation cuvette with an applied voltage of 2.1 kV (MicroPulser Electroporator, Bio-Rad). The spores were immediately resuspended in 1 mL CML (Complete Media Lactose) medium containing 5 g L^–1^ yeast extract, 5 g L^–1^ tryptone and 10 g L^–1^ lactose, and transferred into one well of a 12-well plate. The plate was covered with a breathable sealing film and incubated for 48 h at room temperature on daylight to recover the cells. Finally, the spore suspension was plated in various concentrations onto selective PDA plates containing 100 µg mL^–1^ hygromycin B for antibiotic selection and 0.1% Triton X-100 as colony restrictor. Colonies appeared after 3 days and were further cultivated separately on PDA plates containing 50 µg mL^–1^ hygromycin B and 50 µg mL^–1^ streptomycin (PDA_hyg/strep_ plates).

Transformants were screened in 300-mL Erlenmeyer flasks in 75 mL of a modified MA-medium at 30 °C and 175 rpm for 7 days. The samples were monitored and tested for enzyme activity using the cytochrome *c* activity assay.

### Genomic DNA extraction and colony PCR

Genomic DNA was extracted from a small amount of mycelium collected from a PDA plate. It was put into a screw-cap microfuge tube together with 0.5-mm glass beads and disrupted using a Precellys 24 homogenizer. Three cycles at 5000 rpm were performed for 60 s with suspending for 5 s. Subsequently, 150 µL of 25 mM Tris–HCl buffer, pH 8.0 containing 50 mM glucose and 10 mM EDTA was added and the tube inverted 5 times. It was centrifuged for 1 min at 9500 × g and the supernatant transferred to another tube. For a cleaner sample, the centrifugation was repeated. Five hundred µL of isopropanol was added to the clear supernatant and incubated at -20 °C for 2 h. The DNA precipitate was centrifuged at 9500 × g and 4 °C for 10 min and washed with 4 °C-cold 70% ethanol. The pellet was dried at 60 °C for 30 min and re-suspended in 30 µL purified H_2_O.

ColonyPCR was performed with One*Taq* DNA polymerase from NEB according to the manufacturer’s manual. As forward primer, the sequencing primer (5pLHseq1, Table 4) that attaches in the promoter region was used. The reverse primer (3PcCDH_colPCR, Table 4) was designed to obtain a 300-bp fragment at the beginning of the gene. The PCR was analyzed by gel electrophoresis using 0.8% agarose gel, Gel Loading Dye (6X, NEB) and 2-Log DNA Ladder (NEB) in a horizontal Mini-Sub Cell electrophoresis system (Bio-Rad).

**Table 4:** Nucleotide sequences of primers used in this study

Primer name	Sequence (5′ to 3′)
5pLHseq1	GCCGGCTTCAAAACACACAG
3pLHseq1	CAACATAGCATGTCTTATATATTAAGCC
3PcCDH_colPCR	CCTCTCCGATGAACTCAGTGG

### Production of *Pc*CDH

Expression of *Pc*CDH_*Tr*_ in *T. reesei* was performed in 8 1-L Erlenmeyer flasks in 200 mL modified MA-medium containing 50 µg mL^–1^ streptomycin and 25 µg mL^–1^ chloramphenicol.

Spores were harvested from 8-days-old PDA_hyg/strep_ plates with NaCl-Tween 80 solution (0.9% NaCl, 0.06% Tween 80) and each flask was inoculated to a final spore concentration of 1 × 10^6^ mL^–1^. The cultures were incubated at 30 °C and 160 rpm. Samples were taken, cleared by centrifugation (13,500×*g*, 10 min, 4 °C) and tested for activity using the cytochrome *c* activity assay.

The production of *Pc*CDH by *P. chrysosporium* was performed in 1-L Erlenmeyer flasks placed in an orbital shaker at 130 rpm (2.5 cm eccentricity) and 30 °C. As inoculum, a 1-cm^2^ slab of a five days old culture on PDA was used and homogenized with an Ultra Turrax blender in 250 mL of the medium. The supernatant was harvested after eleven days of growth when exhibiting maximum DCIP activity (130 U L^−1^) and cytochrome *c* activity (130 U L^−1^).

### Purification of *Pc*CDH

The shaking flask cultures were harvested by centrifugation (6500×*g*, 30 min, 4 °C) and vacuum-filtration through a cellulose-filter. The supernatant of each flask was tested for cytochrome *c* activity and pooled for further purification.

As first purification step, hydrophobic interaction chromatography was performed. Solid ammonium sulfate was added to the pooled supernatant to reach 25% saturation and the suspension was cleared from particles by centrifugation (6500×*g*, 30 min, 4 °C) and filtration. The sample was loaded to a Phenyl Sepharose FF hydrophobic column (250 mL, GE Healthcare) equilibrated with 50 mM sodium acetate buffer, pH 5.5 containing 25% (satd.) (NH_4_)_2_SO_4_. A linear gradient from 25 to 0% (NH_4_)_2_SO_4_ in 10 mM sodium acetate buffer (pH 5.5) in 4 column volumes was applied and the protein eluted at 5% (NH_4_)_2_SO_4_. Active fractions were pooled, concentrated and dialyzed against 20 mM MES buffer, pH 6.0 containing 25 mM NaCl using a Vivaflow®50 crossflow module. The sample was applied to a Source 15Q anion exchange column (19 mL, GE Healthcare) equilibrated with the same buffer. The enzymes were eluted with a linear gradient from 25 to 500 mM NaCl in 10 column volumes at a flow rate of 1 mL min^–1^. *Pc*CDH_*Tr*_ eluted at 135 mM NaCl. Fractions were pooled according to cytochrome *c* activity and RZ (A_420_/A_280_) value. The enzyme solution was dialyzed against 50 mM sodium acetate buffer, pH 5.5 and concentrated using a Vivaflow®50 crossflow module with a cut-off of 30 kDa. The molecular weight of the recombinant protein was determined by SDS-PAGE. *Pc*CDH obtained from *P. chrysosporium* cultures was purified with the same procedure and resulted in an enzyme preparation with an RZ-value of 0.59.

### Electrophoretic analysis

SDS-PAGE analysis was carried out by using Mini-PROTEAN TGX Stain-Free precast gels (Bio-Rad) according to the manufacturer’s instructions. For determination of the molecular mass, the Precision Plus Protein Dual Color Standard marker (Bio-Rad) was used. The deglycosylation of recombinant and native CDHs was carried out with Endo H_f_ from NEB according to their protocol.

### MS analysis of glycosylation sites

The samples were digested in solution. The proteins were S-alkylated with iodoacetamide and digested with Trypsin (Promega, Madison, WI). The digested samples were loaded on a BioBasic C18 column (BioBasic-18, 150 × 0.32 mm, 5 µm, Thermo Scientific, Waltham, MA) using 80 mM ammonium formate buffer as the aqueous solvent. A gradient from 5% B (B: 80% acetonitrile) to 40% B in 45 min was applied, followed by a 15-min gradient from 40% B to 90% B that facilitates elution of large peptides, at a flow rate of 6 µL min^–1^. Detection was performed with QTOF MS (Bruker maXis 4G) equipped with the standard ESI source in positive ion, DDA mode (= switching to MSMS mode for eluting peaks). MS-scans were recorded (range: 150–2200 Da) and the 3 highest peaks were selected for fragmentation. Instrument calibration was performed using ESI calibration mixture (Agilent, Santa Clara, CA). The 6 possible glycopeptides were identified as sets of peaks consisting of the peptide moiety and the attached N-glycan varying in the number of HexNAc units, hexose and phosphate residues. The theoretical masses of these glycopeptides were determined with an EXCEL spread sheet using the monoisotopic masses for amino acids and monosaccharides. Manual glycopeptide searches were made using DataAnalysis 4.0 (Bruker, Billerica, MA). Potential glycosylation sites were determined using the online prediction tool provided by the Department of Bio and Health Informatics from the Technical University of Denmark (http://www.cbs.dtu.dk/services/NetNGlyc).

### Spectrophotometry

The FAD occupancy was determined by the method of trichloroacetic acid (TCA) precipitation. TCA was added to 60 µM purified enzyme to a final concentration of 5%. The mixture was heavily mixed for 2 min and cleared by centrifugation (16,000×*g*, 10 min). The supernatant was adjusted to pH 5.5 by adding grains of Na_2_CO_3_. After another centrifugation step, a spectrum of the supernatant was recorded. The amount of FAD was calculated using the molar absorption coefficient for free FAD (ε_450_ = 11.3 mM^–1^ cm^–1^). The CDH concentration was calculated from the absorbance at 280 nm. The molar absorption coefficient of the protein (ε_280_ = 142 mM^–1^ cm^–1^) was determined by the ProtParam tool (https://web.expasy.org/protparam) based on the mature amino acid sequence of *Pc*CDH. The FAD co-factor also contributes to the absorbance at 280 nm. Therefore, the molar absorption coefficient of free FAD at 280 nm (ε_280_ = 20.6 mM^–1^ cm^–1^) was added proportionally to the FAD loading, resulting in a molar absorption coefficient of 156.4 mM^–1^ cm^–1^ for *Pc*CDH_*Tr*_ (70% FAD occupancy) and 162.6 mM^–1^ cm^–1^ for *Pc*CDH (100% FAD occupancy) used for the calculation of the protein concentration.

UV/Vis spectra were recorded from 250 to 700 nm with 70 µM purified enzyme in 20 mM sodium acetate buffer, pH 4.0. For measuring the reduced spectrum, 1 mM cellobiose (final concentration) was added to the enzyme solution.

### Activity assays and steady-state kinetic measurements

The catalytic activity in the expression cultures was monitored photometrically by following cytochrome *c* (ε_550_ = 19.6 mM^−1^ cm^−1^) reduction at 550 nm. The assay was performed in 100 mM sodium acetate buffer, pH 4.5 containing 30 mM lactose as substrate and 20 µM cytochrome *c* as electron acceptor. The reaction was followed for 180 s at 30 °C.

The pH profiles were measured in 100 mM Britton-Robinson buffer (pH range 2.5 – 8) containing 500 µM cellobiose as substrate. Twenty µM of cytochrome *c*, 300 µM of 2,6-dichloroindophenol (DCIP, ε_520_ = 6.9 mM^−1^ cm^−1^), or 500 µM of 1,4-benzoquinone (ε_290_ = 2.24 mM^−1^ cm^−1^) were used as electron acceptors, and the reaction followed at 550, 520, or 290 nm, respectively.

The catalytic constants for cytochrome *c* were determined in 50 mM sodium acetate buffer, pH 4.0 with 500 µM cellobiose as saturating substrate and a cytochrome *c* concentration ranging from 0.125 to 20 µM. Catalytic constants for DCIP were measured in 50 mM sodium acetate buffer, pH 4.5 with 500 µM cellobiose as saturating substrate and a DCIP concentration between 0.5 and 100 µM. The determination of the catalytic constants for electron donors was performed with 300 µM DCIP in 50 mM sodium acetate buffer, pH 4.5. The cellobiose concentration was varied between 10 and 800 µM, lactose was measured between 0.25 and 80 mM and for glucose a range from 0.1 to 2.6 M was used. Catalytic constants were calculated by fitting the measured data to the Michaelis–Menten equation using a non-linear least squares regression in SigmaPlot 12.0 (Systat Software). All measurements were performed in triplicates.

The determination of the reaction rates of mono- and di-substituted 1,4-benzoquinones was performed in 50 mM sodium acetate buffer, pH 4.0 with 0.5 mM cellobiose as substrate and 50 µM of the respective quinone (2,5-dimethoxy-1,4-benzoquinone was measured with 38 µM due to solubility reasons). Five mM stock solutions of 1,4-benzoquinone, methyl-1,4-benzoquinone and methoxy-1,4-benzoquinone were prepared in water. 2,6-Dimethyl-1,4-benzoquinone and 2,6-dimethoxy-1,4-benzoquinone were prepared as 5 mM stock solutions in DMSO, 2,5-dimethoxy-benzoquinone as 1 mM stock solution in DMSO. Their reaction was followed using their respective molar absorption coefficients: ε_246_ = 20.30 mM^–1^ cm^–1^, ε_251_ = 21.45 mM^–1^ cm^–1^, ε_256_ = 15.29 mM^–1^ cm^–1^, ε_257_ = 18.04 mM^–1^ cm^–1^, ε_292_ = 13.68 mM^–1^ cm^–1^, and ε_281_ = 25.96 mM^–1^ cm^–1^ nm.

### Redox potential of *Pc*CDH_*Tr*_

Preparation of CDH-thiol-modified gold electrodes (Ø 3 mm, BAS Inc,) started with mechanical cleaning of the electrode by polishing in an aqueous alumina suspension (Masterprep 0.05 μm, Buehler, DE), rinsed with purified H_2_O and sonicated in purified H_2_O for 10 min to remove residual alumina particles. The electrodes were then subjected to electrochemical cleaning in 0.5 M H_2_SO_4_ for 20 cycles with a scan rate of 200 mV s^−1^, between − 250 and 1700 mV versus Ag/AgCl using cyclic voltammetry, and finally rinsed with purified H_2_O. Thiol pretreatment of the gold electrodes was achieved by immersion in 15 mM 1-thioglycerol in 96% ethanol for 90 min to form a self-assembled monolayer (SAM) on the gold surface. Then, 2 µL of enzyme (5 mg mL^−1^) solution was smeared onto the thiol-modified electrodes. The entrapment of enzyme was achieved by covering the electrode with a dialysis membrane (cut-off 6000–8000 Da) which is fitted tightly to the electrode with a rubber O-ring. The assembly was then sealed with parafilm, leaving out the electrode surface. The electrochemical measurements were performed using an Autolab PGSTAT204 potentiostat (Metrohm). Modified gold electrode, platinum wire, and Ag/AgCl were used as the working-, counter-, and reference (+ 205 mV vs. NHE at 25 °C) electrodes, respectively. All measurements were performed at room temperature in 100 mM potassium-phosphate buffer, pH 6.0 containing 100 mM potassium chloride. The electrolytes of different pH values for midpoint potential measurements were prepared by titration of 1 M NaOH into a universal buffer solution of the following composition: 10 mM acetate, 10 mM phosphate, and 100 mM KCl. The electrolyte was always purged with nitrogen for 15 min prior to the experiment, and a stream of nitrogen was maintained during the measurement.

## Supplementary Information

**Additional file 1:** Vector map of plasmid pLH_*hph*_nat containing the gene sequence of *Pc*CDH under control of the cDNA1 promoter, the hygromycin B and ampicillin resistance gene cassettes, ORI for replication in *E. coli* and a SbfI restriction site for linearization. **Figure S2.** *Pc*CDH amino acid sequence (755 amino acids) with six potential *N*-glycosylation sites highlighted in blue (as predicted by http://www.cbs.dtu.dk/services/NetNGlyc). Positions verified by mass spectrometry are underlined. **Figure S3.** Tryptic digest of *Pc*CDH_*Tr*_ and *Pc*CDH covering the N-glycosylation sequon at position N111. **Figure S4.** Tryptic digest of *Pc*CDH_*Tr*_ and *Pc*CDH covering the N-glycosylation sequon at position N419. **Figure S5.** Tryptic digest of *Pc*CDH_*Tr*_ and *Pc*CDH covering the N-glycosylation sequon at position N434. **Figure S6.** Tryptic digest of *Pc*CDH_*Tr*_ and *Pc*CDH covering the N-glycosylation sequon at position N553. **Figure S7.** Tryptic digest of *Pc*CDH_*Tr*_ covering the N-glycosylation sequon at position N593 and N599.

## Data Availability

All data generated or analyzed during this study are included in this published article and its additional file.
