# Supplementary material for: Heterologous expression of Phanerochaete chrysosporium cellobiose dehydrogenase in Trichoderma reesei
Source: Microb Cell Fact. 2021 Jan 6;20:2. doi: 10.1186/s12934-020-01492-0 (PMC7789494; doi:10.1186/s12934-020-01492-0)
Supplement: Supplementary file 1 — Additional file 1: Vector map of plasmid pLH_hph_nat containing the gene sequence of PcCDH under control of the cDNA1 promoter, the hygromycin B and ampicillin resistance gene cassettes, ORI for replication in E. coli and a SbfI restriction site for linearization. Figure S2. PcCDH amino acid sequence (755 amino acids) with six potential N-glycosylation sites highlighted in blue (as predicted by http://www.cbs.dtu.dk/services/NetNGlyc). Positions verified by mass spectrometry are underlined. Figure S3. Tryptic digest of PcCDHTr and PcCDH covering the N-glycosylation sequon at position N111. Figure S4. Tryptic digest of PcCDHTr and PcCDH covering the N-glycosylation sequon at position N419. Figure S5. Tryptic digest of PcCDHTr and PcCDH covering the N-glycosylation sequon at position N434. Figure S6. Tryptic digest of PcCDHTr and PcCDH covering the N-glycosylation sequon at position N553. Figure S7. Tryptic digest of PcCDHTr covering the N-glycosylation sequon at position N593 and N599. [file 12934_2020_1492_MOESM1_ESM.docx]

**Additional file 1**

**Heterologous expression of *Phanerochaete chrysosporium* cellobiose dehydrogenase in *Trichoderma reesei***

Lena Wohlschlager^1^, Florian Csarman^1^, Hucheng Chang^1^, Elisabeth Fitz^2^, Bernhard Seiboth^2^, Roland Ludwig^1^*

^1^Biocatalysis and Biosensing Laboratory, Department of Food Science and Technology, BOKU – University of Natural Resources and Life Sciences, Muthgasse 18, 1190 Vienna, Austria

^2^Research Division Biochemical Technology, Institute of Chemical, Environmental and Bioscience Engineering, TU Wien, 1060 Vienna, Austria


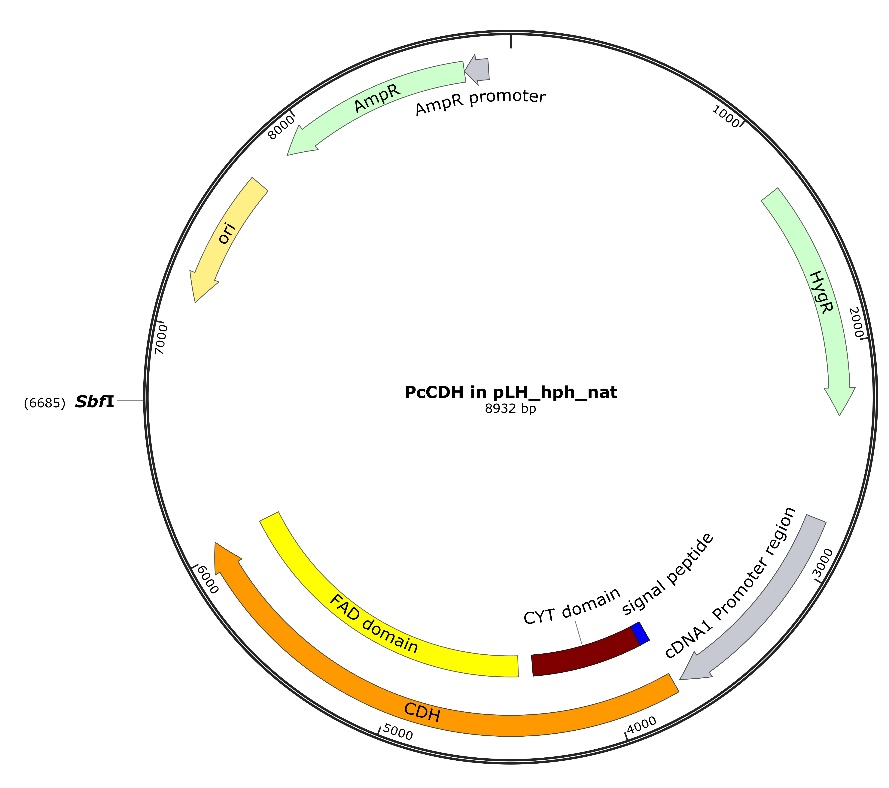


**Figure S1**: Vector map of plasmid pLH_*hph*_nat containing the gene sequence of *Pc*CDH under control of the cDNA1 promoter, the hygromycin B and ampicillin resistance gene cassettes, ORI for replication in *E. coli* and a SbfI restriction site for linearization.

Name: *Pc*CDH sequence Length: 755

QSASQFTDPTTGFQFTGITDPVHDVTYGFVFPPLATSGAQSTEFIGEVVAPIASKWIGIALGGAMNNDLLLVAWANGNQI 80

VSSTRWATGYVQPTAYTGTATLTTLPETTINSTHWKWVFRCQGCTEWNNGGGIDVTSQGVLAWAFSNVAVDDPSDPQSTF 160

SEHTDFGFFGIDYSTAHSANYQNYLNGDSGNPTTTSTKPTSTSSSVTTGPTVSATPYDYIIVGAGPGGIIAADRLSEAGK 240

KVLLLERGGPSTKQTGGTYVAPWATSSGLTKFDIPGLFESLFTDSNPFWWCKDITVFAGCLVGGGTSVNGALYWYPNDGD 320

FSSSVGWPSSWTNHAPYTSKLSSRLPSTDHPSTDGQRYLEQSFNVVSQLLKGQGYNQATINDNPNYKDHVFGYSAFDFLN 400

GKRAGPVATYLQTALARPNFTFKTNVMVSNVVRNGSQILGVQTNDPTLGPNGFIPVTPKGRVILSAGAFGTSRILFQSGI 480

GPTDMIQTVQSNPTAAAALPPQNQWINLPVGMNAQDNPSINLVFTHPSIDAYENWADVWSNPRPADAAQYLANQSGVFAG 560

ASPKLNFWRAYSGSDGFTRYAQGTVRPGAASVNSSLPYNASQIFTITVYLSTGIQSRGRIGIDAALRGTVLTPPWLVNPV 640

DKTVLLQALHDVVSNIGSIPGLTMITPDVTQTLEEYVDAYDPATMNSNHWVSSTTIGSSPQSAVVDSNVKVFGTNNLFIV 720

DAGIIPHLPTGNPQGTLMSAAEQAAAKILALCGGP

**Figure S2:** *Pc*CDH amino acid sequence (755 amino acids) with six potential *N*-glycosylation sites highlighted in blue (as predicted by <http://www.cbs.dtu.dk/services/NetNGlyc>). Positions verified by mass spectrometry are underlined.


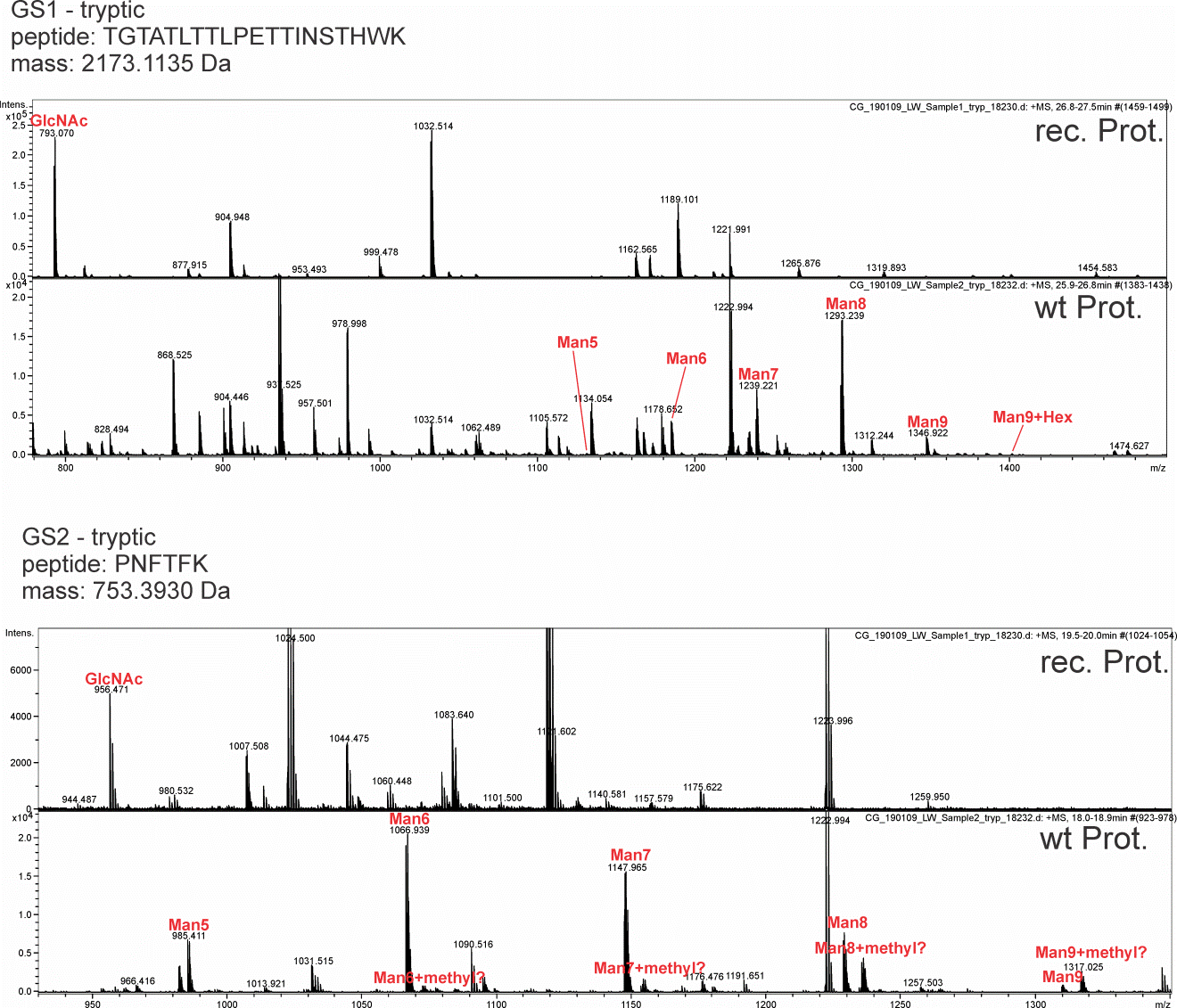


*Pc*CDH*_Tr_*

*Pc*CDH

**Figure S3**: Tryptic digest of *Pc*CDH*_Tr_* and *Pc*CDH covering the N-glycosylation sequon at position N111.


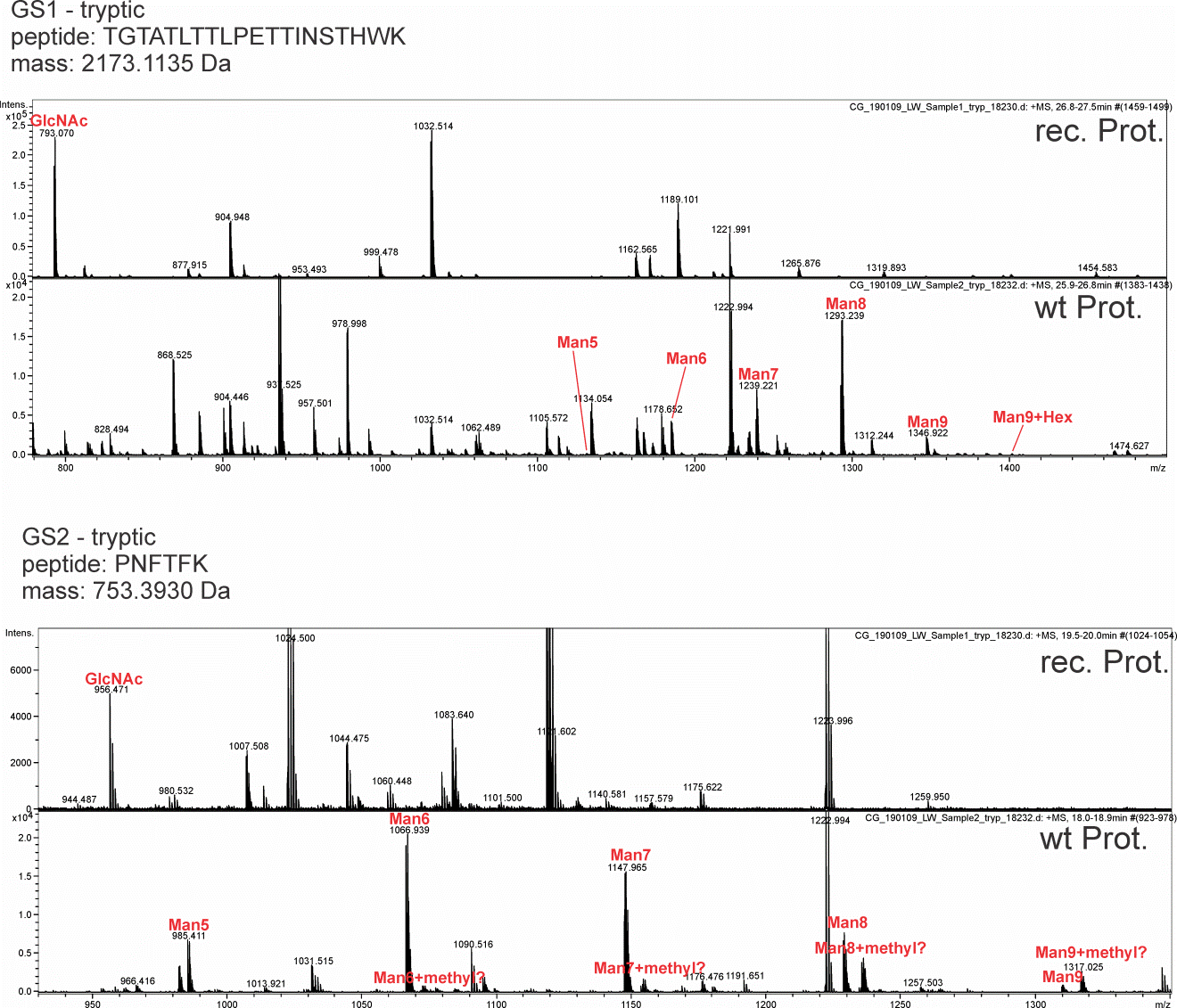


*Pc*CDH*_Tr_*

*Pc*CDH

**Figure S4**: Tryptic digest of *Pc*CDH*_Tr_* and *Pc*CDH covering the N-glycosylation sequon at position N419


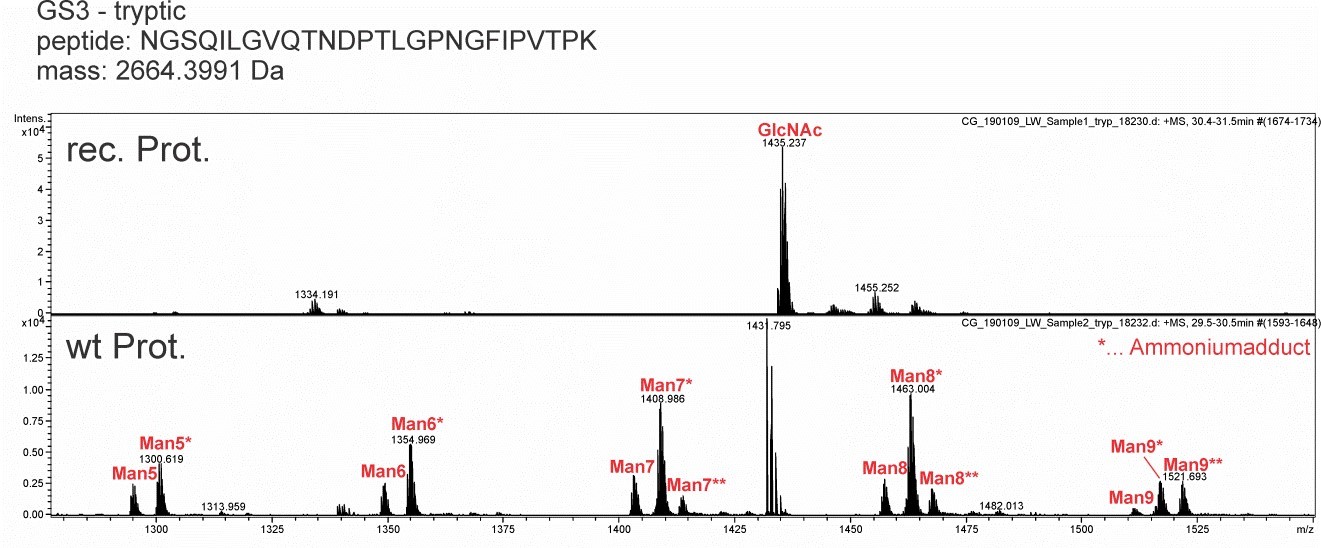


*Pc*CDH*_Tr_*

*Pc*CDH

**Figure S5**: Tryptic digest of *Pc*CDH*_Tr_* and *Pc*CDH covering the N-glycosylation sequon at position N434


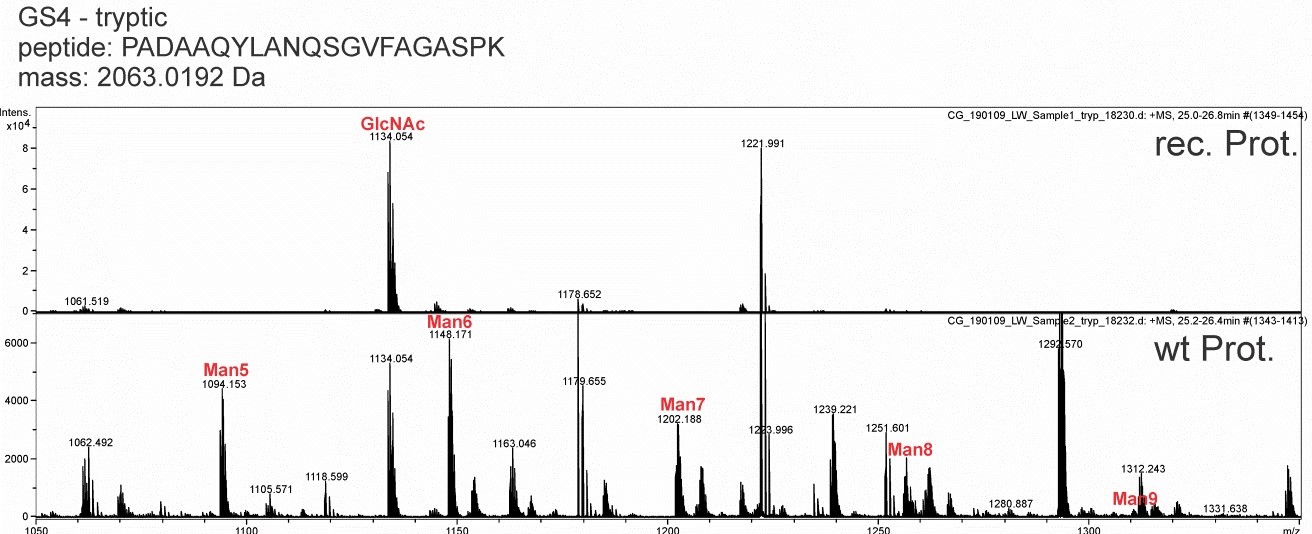


*Pc*CDH*_Tr_*

*Pc*CDH

**Figure S6**: Tryptic digest of *Pc*CDH*_Tr_* and *Pc*CDH covering the N-glycosylation sequon at position N553


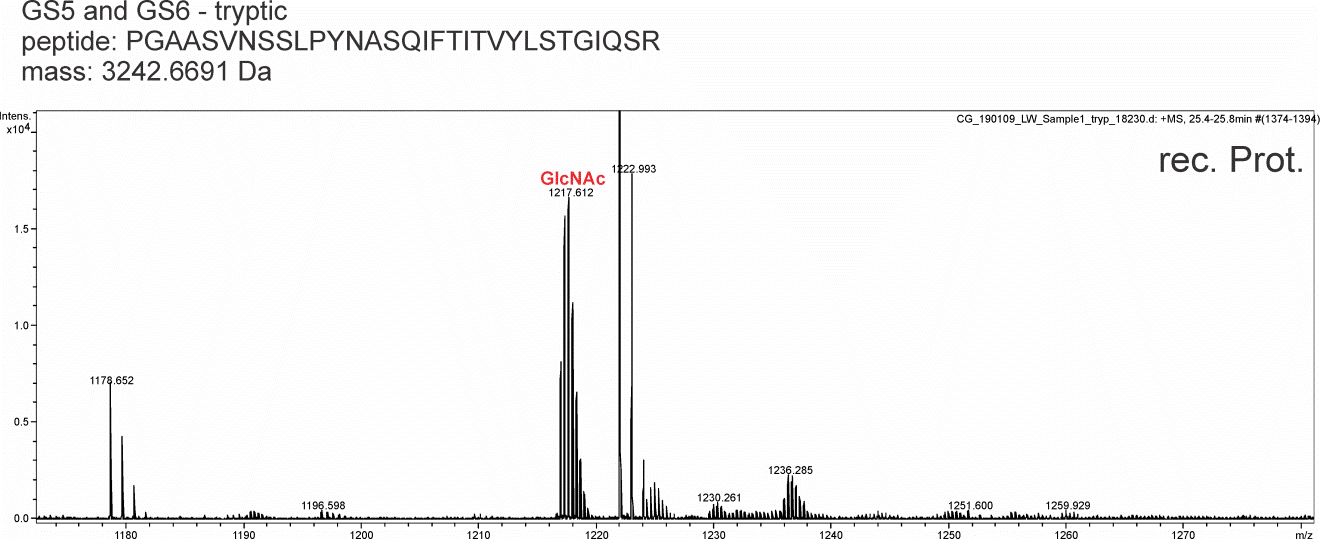


*Pc*CDH*_Tr_*

**Figure S7**: Tryptic digest of *Pc*CDH*_Tr_* covering the N-glycosylation sequon at position N593 and N599
